# Temporal variations of herbage production and nutritive value of three grasslands at different elevation zones regarding grazing needs and welfare of ruminants

**DOI:** 10.5194/aab-62-215-2019

**Published:** 2019-04-17

**Authors:** Maria Koidou, Ioannis Mountousis, Vassilios Dotas, Konstantinos Zagorakis, Maria Yiakoulaki

**Affiliations:** 1 Department of Animal Production, School of Agriculture, Aristotle University of Thessaloniki, Faculty of Agriculture, Forestry and Natural Environment, 54124 Thessaloniki, Greece; 2 Department of Animal Production, Faculty of Agriculture, Technological Educational Institute of Western Macedonia, Terma Kontopoulou 53100 Florina, Greece; 3 Hellenic Agricultural Organization “Demeter”, Research Institute of Animal Science, 58100 Giannitsa, Greece; 4 Department of Range and Wildlife Science, School of Forestry and Natural Environment, Faculty of Agriculture, Forestry and Natural Environment, Aristotle University of Thessaloniki, 54124 Thessaloniki, Greece

## Abstract

Interannual and monthly variations of herbage production and nutritive value regarding grazing ruminants' needs and welfare were evaluated in three grasslands (semi-mountainous, mountainous and sub-alpine) located at different altitudes (480–900, 901–1500 and 1501–2334 m, respectively) in northern Greece during 2015–2016. Herbage biomass was collected from 30 experimental cages (10 per grassland), weighed, dried at 65 ${}^{\circ }$C, milled and analyzed for crude protein (CP), neutral detergent fiber (NDF), acid detergent fiber (ADF), lignin, calcium (Ca) and phosphorus (P) content and in vitro dry matter digestibility (IVDMD). The sub-alpine grassland was the most productive (1031 and 1231 kg DM ha${}^{-1}$) with the highest mean annual herbage CP content (93 and 87 g kg${}^{-1}$ dry matter; DM) for 2015 and 2016, respectively. CP content was sufficient to meet small ruminants' and beef cattle maintenance requirements until April and May in the semi-mountainous and mountainous grasslands, respectively, while it could cover the above requirements in the sub-alpine grassland until June and August, respectively. The herbage Ca concentration was higher than the grazing ruminants' needs, while the phosphorus concentration was insufficient. Protein and phosphorus supplementation should be provided to animals to cover their maintenance requirements during the whole period and to reach high levels of welfare. Even though grazing is considered as a welfare-friendly procedure, it is uncertain whether all the welfare principals are satisfied in extensive production systems due to variations of forage availability and nutritive value as well as the lack of infrastructure in grasslands.

## 1 Introduction

Native grasslands in Greece occupy an area of 5.45 million ha corresponding to 41.3 % of the total rangelands in the country (Hellenic Statistical Authority, 2000). They are multifunctional areas as they provide goods and ecosystem services, such as the maintenance of biodiversity, feed and shelter to the game animals, landscape–soil–air and water quality, recreation, rural employment, cultural and social benefits (Papanastasis and Ispikoudis, 2012). However, the main use of grasslands is the grazing by all kind of livestock, mostly sheep and beef cattle. The large part of Greek grasslands (75 %) belongs to the state and they are used communally for a low-input livestock production system. Communal use of grasslands means that the owner of each flock/herd can freely utilize the grasslands (uncontrolled grazing) that are allocated to the place where he resides (Yiakoulaki and Papanastasis, 2014).

Grasslands are characterized by spatial and temporal variability of climatic conditions, variations in altitude, topography and soils, as well as high species richness (Papanastasis and Mansat, 1996; Karagiannakidou et al., 2001). Their productivity is relatively low and prone to seasonal variations, which results to the creation of two feed gaps: a short gap in winter (about 2 months) and a longer one (about 4 months) in the summer. Also, there is an interannual variability in the forage production of grasslands which could potentially increase up to 3 times in the wet years. The farmers depend greatly on these ecosystems to raise their animals. To counterbalance the sort feed gap, they utilize temporary pastures with annual winter cereals (e.g., barley, wheat, rye and oats; mainly as monocultures), while in order to fill the long feed shortage, they lead their livestock for grazing to sub-alpine grasslands, practicing transhumance. The latter is also a common strategy used by farmers in several areas of Europe (Hopkins, 2011), so that animals can take advantage of the better quantity and quality of herbage during this period. However, due to socioeconomic reasons, transhumance has significantly declined over the last decades (Yiakoulaki and Papanastasis, 2014).

Every natural grassland has a unique composition of plant species differing in morphological characteristics and stage of development. This heterogeneity makes it difficult to characterize the seasonal dynamics of herbage production and nutritive value. The abovementioned variations are considered to be among the most important constraints of livestock production (Ngwa et al., 2000) and their evaluation is essential in extensive (grazing-based) production systems. Grazing affects the quantity and quality of herbage in grasslands (Zhai et al., 2018) and subsequently the behavior and welfare of free-ranging livestock (Kilgour et al., 2012). Animals during grazing are able to select the preferred forage species and to avoid the toxic ones, express their natural behavior and maintain their health (Villalba et al., 2010; Charlton and Rutter, 2017). However, in extensive production systems, problems concerning the animals' welfare maybe arise from several issues, such as decreased forage availability, forage nutrient deficiencies, lack of access to water and shelter, inappropriate stable installations, inadequate veterinarian care, etc. Research about the welfare of livestock has been mainly conducted to intensively farmed animals with or without access to pastures (Turner and Dwyer, 2007; Charlton and Rutter, 2017; Carcangiu et al., 2018) and to certain regions (Mikuš et al., 2018). For this reason, information concerning the welfare of grazing ruminants is quite restricted (Bojkovski et al., 2014; Topczewska, 2014; Antkowiak et al., 2012; Karasabbidis et al., 2014; Gilhaus and Hölzel, 2016).

The objectives of this study were (a) to determine the interannual and monthly variations of forage production in three grasslands located at different altitudinal zones, (b) to estimate the interannual and monthly variations of herbage nutritive value and to relate them to the nutritional needs of grazing ruminants and (c) to discuss issues regarding the animal's welfare in extensive production systems.

## 2 Materials and methods

### 2.1 Study area

The study was conducted in three grasslands in the Florina Prefecture in Western Macedonia, Greece (latitude 40${}^{\circ }$77${}^{\prime }$ to 40${}^{\circ }$89${}^{\prime }$ N, longitude 21${}^{\circ }$70${}^{\prime }$ to 21${}^{\circ }$49${}^{\prime }$ E) from March to November 2015 and 2016. They are located in the same area but in different elevation zones, with an altitude ranging from 480 to 2334 m a.s.l.: semi-mountainous grassland (480–900 m), mountainous grassland (901–1500 m) and sub-alpine grassland (1501–2334 m). Metamorphic rock textures (i.e., phyllites) of the Pelagonic geotectonic zone are the dominant geological substrates of the study area (Koroneos, 1991) and the soils were classified as acid sandy loam according to Bouyoucos (1962). The climate is not the typical Greek Mediterranean (Papanikolaou et al., 2002) but it approaches the middle-European type (Papanastasis, 1982), reaching in winter even -20 ${}^{\circ }$C (H. N. M. S., 2017). The mean annual and monthly rainfall and air temperature during the experimental period (2015 and 2016) are shown in Table 1. According to Flocas (1994), the average air temperature decreases about 0.6 ${}^{\circ }$C per 100 m from the lower to the higher elevation zone. This reduction is more intensive in summer compared to winter.

**Table 1:** Mean annual and monthly values of rain precipitation (mm) and air temperature (${}^{\circ }$C) during the experimental period.

Environmental	March	April	May	June	July	August	September	October	November	Mean ± SD
conditions										
Air temperature (${}^{\circ }$C)										
2015	5.01	10.25	17.05	18.94	24.03	22.24	19.17	12.49	9.79	15.44±6.20
2016	7.80	14.40	15.00	21.05	23.00	21.60	16.95	12.20	6.55	15.39±5.74
Rain precipitation (mm)										
2015	104.31	37.51	15.84	46.61	22.35	90.05	129.39	114.94	17.28	64.21±46.16
2016	85.72	16.43	103.02	67.12	36.92	41.78	141.42	34.46	44.16	63.41±39.79

Source: Florina and Amyntaio's weather stations (H. N. M. S., 2017); SD: standard deviation.

The topography combined with the variable climatic conditions from the semi-mountainous to the sub-alpine area creates an impressive variety of flora to the grasslands. Flat areas and openings with a great variety of herbaceous plants in oak forests (*Ostryo*-*Carpinion orientalis* and *Quercion frainetto*) form the semi-mountainous grassland. *Dichanthium ischaemum, Dactylis glomerata, Festuca heterophylla, Briza maxima, Agrostis tenuis, Cynodon dactylon, Poa nemoralis, Festuca rubra, Cynosurus echinatus, Bromus squarrosus, Poa bulbosa, Trifolium repens, Trifolium subterraneum, Trifolium angustifolium, Medicago lupulina, Medicago sativa, Anthyllis aurea, Astragalus glycyphyllos* and *Lathyrus laxiflorus* were the main herbaceous species in this grassland.

The mountainous grassland is characterized by oak and mainly beech trees (*Quercion frainetto* and *Fagion moesiacae* zone) in the overstorey vegetation. *Alopecurus pratensis, Bromus benekenii, Festuca arundinacea, Poa nemoralis, Briza media, Dactylis glomerata, Melica ciliata, Anthoxanthum odoratum, Poa compressa, Brachypodium sylvaticum, Trifolium alpestre, Trifolium repens, Trifolium subterraneum, Medicago lupulina, Coronilla varia, Vicia grandiflora, Astragalus depressus* and *Teucrium chamaedrys* were the dominant herbaceous species in the understory vegetation.

The sub-alpine grassland is covered by perennial herbaceous plant species such as *Festuca peristerea, Festuca horvatiana, Poa media, Secale montanum, Avenula pubescens, Phleum hirsutum, Stipa epilosa, Avena pratensis, Bellardiochloa violacea, Poa molineri, Trifolium pratense, Trifolium alpestre, Trifolium badium, Vicia onobrychioides, Valeriana officinalis, Thymus thracicus, Teucrium montanum, Veronica praecox, Potentilla micrantha* and *Saxifraga rotundifolia.*

### 2.2 Husbandry system

The husbandry system in the study area is mainly based on traditional grazing of communal grasslands which provide herbage to animals for 8–9 months of the year. During winter, due to the low temperatures, the animals are kept in the stables, and feedstuffs (roughage and concentrates) are provided to them. The animals graze in the semi-mountainous and mountainous grasslands from spring to autumn while in summer the grazing starts in the sub-alpine grasslands. The dominant livestock species in the study area were sheep (79 306) followed by goats (14 336) and cattle (13 181). Mean stocking density was 0.93, 0.08 and 0.30 LU ha${}^{-1}$ in the mountainous, semi-mountainous and sub-alpine grasslands, respectively. The equivalent livestock units (LUs) were calculated as 1 LU: cows older than 24 months; 0.6 LU: cows between 6 and 24 months; and 0.2 LU: cows younger than 6 months. For sheep and goats, 1 LU is equivalent to 0.15 breeding females and 0.12 adult males. Flocks were pure or mixed and shepherded for the whole year. Sheep and goats are raised for milk and meat, while cattle are raised for meat.

### 2.3 Sampling and experimental analyses

A total of 10 experimental cages of 16 m${}^{2}$ were placed at selected points on each grassland (30 cages in total) before the beginning of the vegetation measurements in 2015. Each cage was fenced with a 1.5 m height metal mesh net in order to prevent the free grazing from livestock. The cages were divided into 16 equal plots of 1 m${}^{2}$. Herbage biomass was collected at the beginning of each month, from one plot of each cage every time. The herbage samples (the woody species were excluded if present) were clipped at 2 cm above the soil surface using hand scissors. The harvested herbage samples were handled in order to avoid soil contamination. Also, plant parts of previous years, that are not preferred by grazing animals, were removed. Samples were placed in paper bags, instantly weighed in the field to obtain the measure of fresh-cut herbage weight and expressed as kilograms of dry matter per hectare (kg DM ha${}^{-1}$). All samples were dried in the oven at 65 ${}^{\circ }$C until constant weight and milled using the Cross Beater Mill SK 100 (Retsch, Germany, 1 mm screen). Then, they were divided into two subgroups and stored in glass vases.

Samples from each subgroup were used for the chemical analyses in triplicates. They were analyzed for neutral detergent fiber (NDF), acid detergent fiber (ADF), lignin, crude protein (CP), calcium (Ca) and phosphorus (P) content as well as for in vitro dry matter digestibility (IVDMD). Neutral detergent fiber was determined according to the method of Van Soest et al. (1991). Samples were analyzed using heat-stable α-amylase (A3306, Sigma–Aldrich, St. Louis, MO, USA) without sodium sulfite in the neutral detergent reagent. Acid detergent fiber (ADF) was determined according to AOAC (2000a; method 973.18). Both NDF and ADF values were expressed without residual ash. Lignin concentration was determined by the sulfuric acid procedure (AOAC, 2000a; method 973.18) and ash was determined by the gravimetric residue after heating to 550 ${}^{\circ }$C for 8 h. Nitrogen (N) content was determined using copper as a catalyst according to the Kjeldahl method (AOAC, 2000b; method 984.13) and crude protein was calculated as N ×6.25.

The IVDMD was determined according to the two-stage technique of Tilley and Terry (1963) using the following equation:

1 $$\mathrm{IVDMD}=\left(\frac{1-w_{d}-w_{b}}{w_{s}}\right)\times 100,$$

where $w_{d}$ is the weight of dry plant sample residue, $w_{b}$ is the weight of dry residues of blank, and $w_{s}$ is the dry weight of the original plant sample.

Concentrations of calcium (Ca) and phosphorus (P) in the herbage samples were measured by oxidizing each subsample with a 2:1 nitric / perchloric acid mixture. In separate aliquots, Ca was determined by flame photometry and P by spectrophotometric methods (Khalil and Manan, 1990). All the presented values in the study were means of six measurements (three replicates × two subsamples = six measurements).

### 2.4 Statistical analysis

The data of herbage production, nutritive value (CP, NDF, ADF, acid detergent lignin (ADL) and IVDMD) and mineral content (Ca and P) were analyzed with the least-squares method (LSM) analysis in SPSS v.23 software for Windows (SPSS Inc., Ill: Chicago, USA) (Kitikidou, 2005) according to the following model:

2 $$yijk=\mu +a_{i}+b_{j}+c_{k}+abc_{ijk}+e_{ijkm},$$

where $y_{ijk}$ is herbage production, CP, NDF, ADF, ADL, IVDMD, Ca and P content, μ is the overall mean, $a_{i}$ is the constant effect of the altitudinal zone of the three grasslands (i=1,2,3), $b_{j}$ is the constant effect of the month (j= I, II, … IX), $c_{k}$ is the constant effect of the sampling year (k=1,2), $abc_{ijk}$ is the constant effect of the i-altitudinal zone of the three grasslands with j the month, k the year and $e_{ijkm}$ the random residual effect.

The impact of altitudinal zone on herbage production and nutritive value was estimated using Fisher's protected least significant difference (LSD) test (Fisher, 1966). The significance level was set at P<0.05.

## 3 Results

### 3.1 Herbage production

**Figure 1:**
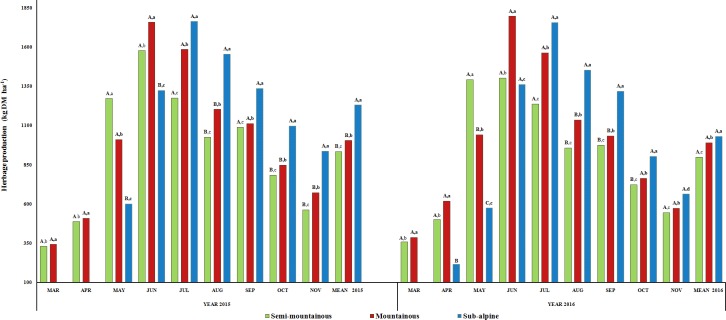
Interannual and monthly variation of herbage production (kg DM ha${}^{-1}$) of the semi-mountainous, mountainous and sub-alpine grasslands in the Florina Prefecture, northern Greece. Means of the three grasslands between the 2-year experimental period followed by different letters (a–d) along the corresponding bar differ at P<0.05. Means of the three grasslands in the same year of the experimental period followed by different capital letters (A–C) along the corresponding bar differ at P<0.05.

The mean annual production of herbaceous species was higher (P<0.05) in the sub-alpine grassland compared to the other two grasslands in 2015, while no significant differences (P>0.05) were detected between the mountainous and semi-mountainous grasslands (Fig. 1). In 2016, the mean annual herbage production of the sub-alpine grassland was greater than the other two grasslands but with no significant differences (P>0.05) between them. Also, there were no interannual differences (P>0.05) for the herbage production of each grassland. The herbage production of all grasslands followed the same trend throughout the growing period in both years. Specifically, it increased from spring to midsummer and then decreased until the end of autumn. Peak production was recorded in the semi-mountainous and mountainous grasslands during June, while in the sub-alpine grassland it occurred in July. The herbage production of the three grasslands was significantly affected (P<0.001) by the harvest month, the altitudinal zone (P<0.05) and by the “month x altitudinal zone” interaction (P<0.001), as presented in Table 2.

**Table 2:** Effect of harvest time (month and year) and altitudinal zone on herbage production and nutritive value.

Parameter	Month	Zone	Year	Month	Month	Zone	Month x zone
				x zone	x year	x year	x year
Herbage production	${}^{***}$	${}^{*}$	NS	${}^{***}$	NS	NS	NS
CP	${}^{***}$	${}^{***}$	${}^{**}$	${}^{***}$	NS	NS	NS
NDF	${}^{***}$	${}^{***}$	${}^{***}$	${}^{***}$	${}^{***}$	${}^{***}$	${}^{***}$
ADF	${}^{***}$	${}^{***}$	NS	${}^{***}$	${}^{***}$	${}^{**}$	${}^{**}$
ADL	${}^{***}$	${}^{***}$	NS	${}^{***}$	NS	NS	${}^{*}$
IVDMD	${}^{***}$	${}^{***}$	NS	${}^{***}$	${}^{***}$	${}^{***}$	${}^{*}$
Ca	${}^{***}$	${}^{***}$	${}^{***}$	${}^{***}$	${}^{***}$	${}^{**}$	${}^{***}$
P	${}^{**}$	${}^{***}$	${}^{***}$	NS	NS	${}^{*}$	NS

CP: crude protein; NDF: neutral detergent fiber assayed with a heat-stable amylase and expressed free of residual ash; ADF: acid detergent fiber; ADL: acid detergent lignin; IVDMD: in vitro dry matter digestibility; Ca: calcium; P: phosphorus. Level of significance: ${}^{***}$: P<0.001; ${}^{**}$: P<0.01; ${}^{*}$: P<0.05; NS: not significant.

### 3.2 Crude protein, cell wall contents and in vitro digestibility

**Figure 2:**
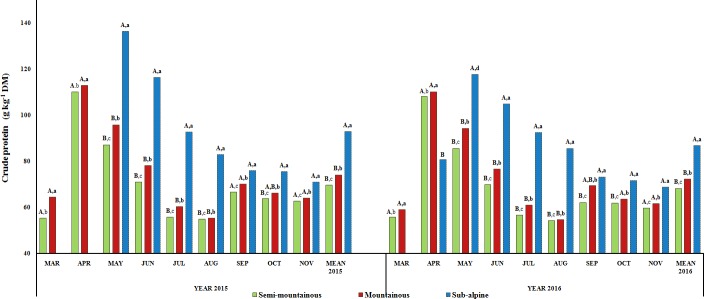
Interannual and monthly variation in herbage crude protein (CP; g kg${}^{-1}$ DM) content of the semi-mountainous, mountainous and sub-alpine grasslands in the Florina Prefecture, northern Greece. Means of the three grasslands between the 2-year experimental period followed by different letters (a–d) along the corresponding bar differ at P<0.05. Means of the three grasslands in the same year of the experimental period followed by different capital letters (A–C) along the corresponding bar differ at P<0.05.

Over the 2-year study, the effects of month, altitudinal zone, year and “month x zone” interaction (P<0.001; P<0.01) on herbage CP content were revealed (Table 2). The mean annual CP content of herbaceous species was higher (P<0.05) in the sub-alpine grassland compared to the two other grasslands in both years, while there were no significant differences (P>0.05) between the mountainous and semi-mountainous grasslands (Fig. 2). Similarly, no interannual differences were detected (P>0.05) for the herbage CP content of each grassland. Regarding the monthly variations of herbage CP content of semi-mountainous and mountainous grasslands, they increased from March to April and decreased until August. A second increase of the CP content was observed during September, reaching its lower value in November of both years. On the other hand, the herbage of the sub-alpine grassland initiated growth during late spring when the CP content was high. Then, it decreased until the end of the growing season.

**Figure 3:**
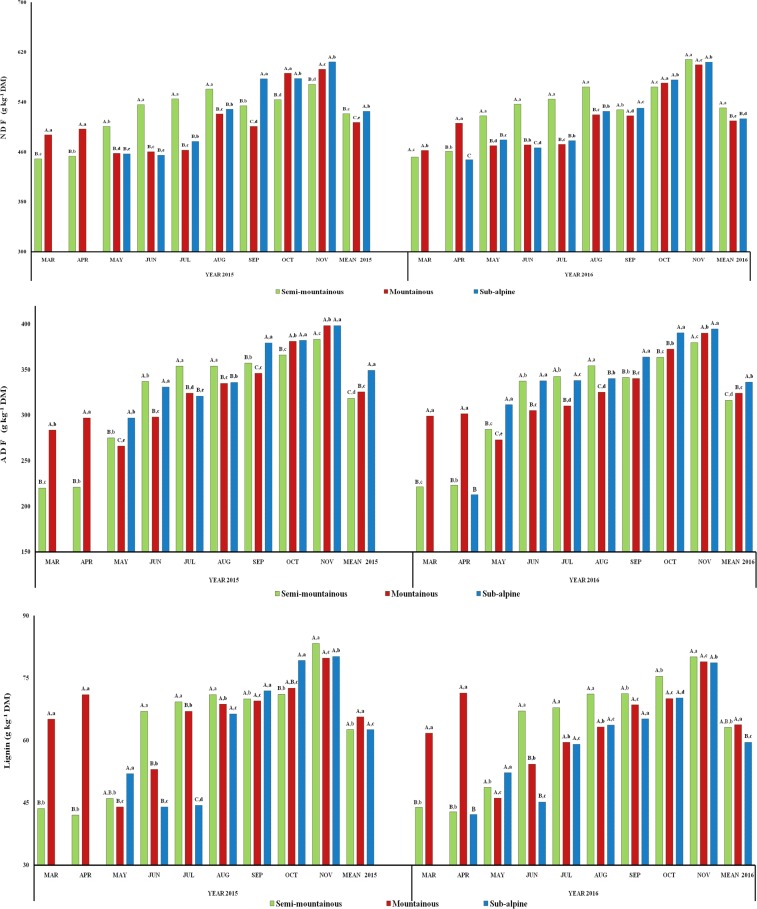
Interannual and monthly variation of herbage cell wall constituents (NDF, ADF, ADL; g kg${}^{-1}$ DM) of the semi-mountainous, mountainous and sub-alpine grasslands in the Florina Prefecture, northern Greece. Means of the three grasslands between the 2-year experimental period followed by different letters (a–d) along the corresponding bar differ at P<0.05. Means of the three grasslands in the same year of the experimental period followed by different capital letters (A–C) along the corresponding bar differ at P<0.05.

The herbage NDF content of the three grasslands was significantly affected (P<0.001) by month, zone, year and their interactions, while the ADF content was not affected by year (P>0.05). A significant effect of month and zone as well as of interactions of month x zone (P<0.001) and month x zone x year (P<0.05) was found for the ADL content of herbage. The cell wall constituents increased as the growing season progressed, showing a peak value during November in both years (Fig. 3). The annual mean NDF concentration of herbage was significantly different (P<0.05) among the three grasslands in 2015, but in 2016, it was higher (P<0.05) in the semi-mountainous grassland with no significant differences (P>0.05) between the two other grasslands. Interannual differences were also detected (P<0.05) for the herbage NDF of the semi-mountainous and sub-alpine grasslands. The annual mean herbage ADF value of the sub-alpine grassland was higher (P<0.05) than the two other grasslands in both years. Interannual differences (P<0.05) for the herbage ADF content were detected only in the sub-alpine grassland. The annual mean ADL content was not significantly different (P>0.05) in the three grasslands in 2015, while it was lower (P<0.05) in the sub-alpine grassland in 2016. Interannual differences of the herbage ADL content were not detected (P>0.05) in each grassland, while the monthly ADL content of the three grasslands increased from the beginning to the end of the experimental period.

**Figure 4:**
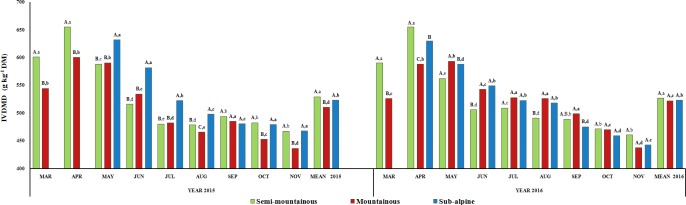
Interannual and monthly variation of herbage in vitro dry matter digestibility (IVDMD; g kg${}^{-1}$ DM) of the semi-mountainous, mountainous and sub-alpine grasslands in the Florina Prefecture, northern Greece. Means of the three grasslands between the 2-year experimental period followed by different letters (a–d) along the corresponding bar differ at P<0.05. Means of the three grasslands in the same year of the experimental period followed by different capital letters (A–C) along the corresponding bar differ at P<0.05.

The annual mean in vitro dry matter digestibility of herbaceous species was lower (P<0.05) in the mountainous grassland in 2015 compared to the two other grasslands, while there were no differences (P>0.05) among the three grasslands in 2016 (Fig. 4). Interannual differences (P<0.05) for the herbage IVDMD were detected only for the mountainous grassland. In the semi-mountainous grassland, the herbage IVDMD increased from March to April and then showed a progressive reduction until the end of the experimental period in both years. The herbage IVDMD of the mountainous grassland followed the same trend but its peak value was observed in May 2016. On the other hand, the herbage IVDMD of the sub-alpine grassland was slowly decreased from the beginning to the end of the experimental period by 26 % and 28  % in 2015 and 2016, respectively. Statistical analyses showed that IVDMD of herbaceous species was significantly affected (Table 2) by harvest time (only by month), altitudinal zone and their interactions (P<0.001; P<0.05).

### 3.3 Mineral concentrations

**Figure 5:**
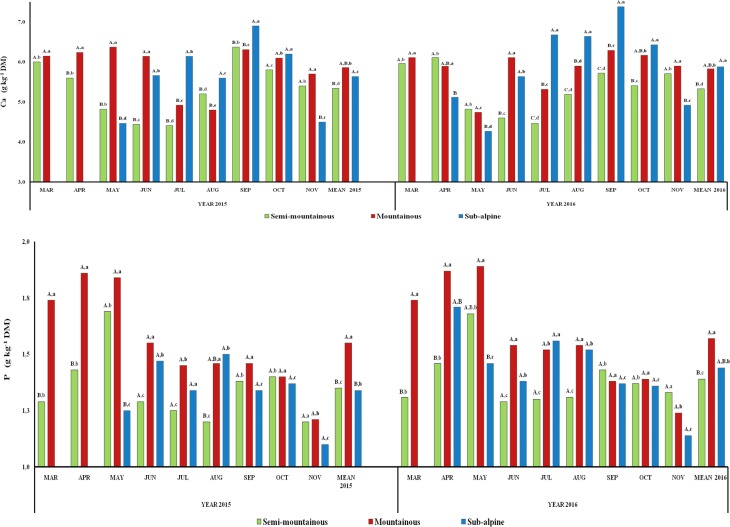
Interannual and monthly variation of herbage calcium and phosphorus contents (g kg ${}^{-1}$ DM) of the semi-mountainous, mountainous and sub-alpine grasslands in the Florina Prefecture, northern Greece. Means of the three grasslands between the 2-year experimental period followed by different letters (a–d) along the corresponding bar differ at P<0.05. Means of the three grasslands in the same year of the experimental period followed by different capital letters (A–C) along the corresponding bar differ at P<0.05.

The annual mean calcium concentration of the herbaceous species was significantly different (P<0.05) between the semi-mountainous and sub-alpine grasslands both in 2015 and 2016 (Fig. 5). Interannual differences (P<0.05) were detected only in the sub-alpine grassland. Monthly trends of herbage Ca concentration were also observed in all three grasslands with an increase during September. Herbage Ca concentration was affected significantly (P<0.001) by the harvest time (month and year) and altitude zone, as well as their interactions (P<0.001; P<0.01) (Table 2).

The annual mean P concentration of herbage was significantly higher (P<0.05) in the mountainous grassland compared to the two other grasslands in 2015, while in 2016 significant differences (P<0.05) were found between the semi-mountainous and mountainous grasslands. Interannual differences were not found (P>0.05) among the three grasslands (Fig. 5). The P concentration in the semi-mountainous and mountainous grasslands was higher in the summer months in both years, while in the sub-alpine grassland the peak occurred in August and April of 2015 and 2016, respectively. The phosphorus concentration was affected significantly (P<0.001) by the altitudinal zone of grasslands and the sampling year (Table 2) as well as by the harvest month (P<0.01).

## 4 Discussion

Information about monthly and interannual variations of forage production and nutritive value is needed in order to achieve a time-efficient utilization of forage in grasslands and to detect nutrient deficiencies of grazing animals. This will lead to the administration of the appropriate supplementation and to high levels of grazing animals' welfare. The discussion below follows this line of thought.

### 4.1 Herbage production

The herbage production in the mountainous grasslands varies greatly as it is affected by the climatic conditions (mainly temperature and precipitation), the altitudinal zone, the botanical composition of plant species, the livestock grazing and the management practices (Papanastasis, 1982; Smith et al., 2008; Mpokos et al., 2014). It has been reported by Papanastasis (1982) that, in dry and semi-dry areas of Greece, annual herbage production of grasslands is usually below 1500 kg ha${}^{-1}$, while in subhumid and humid areas it may reach 3000–4000 kg ha${}^{-1}$. These differences are also reflected in the altitudinal zone, with grasslands at the lowlands having lower productivity than that of the higher ones. In our study, the herbage production for all three grasslands is relatively low. The sub-alpine grassland was the most productive grassland with mean annual values of 1031 and 1231 kg DM ha${}^{-1}$ for 2015 and 2016, respectively. However, these findings are also lower compared to the results reported by other researchers (Stoliou, 2011; Mpokos et al., 2014) for other sub-alpine grasslands in northern Greece. This could be attributed to the fact that the vegetation of previous years was removed during the sampling process of our study and forage production represented only the annual growth of herbaceous species. Apart from the effect of altitude, air temperature plays an important role in the amount of biomass produced. However, the beginning and the end of the plant growth period are determined by the rainfall (George et al., 2001). As presented in Fig. 1, no samples were collected from the sub-alpine grassland during March and April 2015 and March 2016 as a result of snow coverage. The seasonal snow cover in mountainous areas has a direct effect on local climate and hydrology as well as on the functioning of ecosystems (Pelto, 2008). By acting as frozen storage in the water balance, the snowpack provides an important source of freshwater recharge in the spring, influencing runoff, soil moisture and groundwater (Barnett et al., 2005). The impact of rising temperature on the reduction of the mountain snowpack resulted in more water available to plants and consequently greater herbage production in mountainous and sub-alpine grasslands during the summer. Herbage production tended to increase until midsummer for the semi-mountainous and mountainous grasslands, and until the end of summer for the sub-alpine one, and then decreased as the growing period progressed in both years introducing, however, a sigmoid curve similar to the findings reported by Pearson and Ison (1987). A similar pattern for herbage production was also found by Mountousis et al. (2008) on mountainous and sub-alpine grasslands in NW Greece as well as by Stoliou (2011) in the sub-alpine pastures of Greek Voras and Olympus mountains.

### 4.2 Crude protein, cell wall contents and in vitro digestibility

The herbage crude protein concentration of all three grasslands was higher at the beginning of the growth period (Fig. 2), as a result of the intense growth of plants (Ryan and Bormann, 1982). As the season progressed and plants matured, the CP content decreased due to the increased proportions of structural carbohydrates vs. cell contents and the accumulation of nutrients to flowers and seeds. Also, the occurred changes in the stem / leaf ratio contributed to the total CP reduction as stems contain less crude protein than leaves (Sheaffer et al., 1992). An increase in the herbage CP content was observed in the semi-mountainous and mountainous grasslands during September. Rain and cooler temperatures during this month initiated a second growth cycle of plants, at which time the CP content was increased. However, this trend was not observed in the sub-alpine grassland, where the CP content steadily declined after May by 48 % and 41.5 % in 2015 and 2016, respectively. This probably indicates that the perennial plant species that compose the sub-alpine grassland seem not to be responding enough to climatic changes for second growth cycle, and consequently an increment in the CP content, but they go dormant in preparation for winter. In general, information concerning the regrowth cycle of herbaceous species is very limited (Michaud et al., 2011) and there are no published data to compare with our results. Mean CP content of herbage was higher in the sub-alpine grassland compared to the two other grasslands, thus reflecting the climatic conditions that occurred during the growth period of plants (Holecheck et al., 2010). Moreover, according to Han et al. (1997), plants growing at higher altitudes improve their resistance to cold environments by increasing the content of substances like CP, fat, starch and sugars (sucrose, fructose and glucose).

Crude protein is an essential dietary nutrient for animals' maintenance, growth and reproduction. The CP requirements for maintenance of livestock range from 82 g kg${}^{-1\;}$DM for growing beef cattle (with a live weight of 300 kg and an average daily gain of 0.22 kg) to 95 g kg${}^{-1}$ DM for small ruminants (sheep and goats) with a live weight of 50 kg, according to NRC (1985, 1996). In the studied grasslands, beef cattle requirements for the CP could be adequately covered from April to May in the semi-mountainous and mountainous grasslands, while in the sub-alpine grassland they are covered from May to August. The CP requirements of small ruminants could be adequately satisfied only at the beginning of the grazing season (April) in the semi-mountainous and mountainous grasslands and from May to June in the sub-alpine grassland. For the rest period, additional protein sources should be supplied in order to meet the maintenance requirements of the animals. Additionally, Mountousis et al. (2011) suggested that the move of grazing animals from the lowlands to high-elevation grasslands during summer seems to be a good practice in order to take advantage of herbage with a better crude protein content.

The herbage NDF, ADF and lignin constituents were low in the early vegetative stages of the three grasslands and increased as the growing period progressed, showing their peak value at the end of autumn. The fiber concentration increased and the nutrient content decreased with plants' maturation, affecting their digestibility and nutritive value as well (Buxton and Redfearn, 1997; Feyissa et al., 2014). In our study, the fiber content of herbage in all grasslands followed almost this pattern, albeit with different intensities. The higher cell wall contents were found in the semi-mountainous grassland, as a result of the early maturation of plants due to the higher temperature and lower humidity, especially during summer. This is in accordance with the results of Roukos et al. (2011) for grasslands in northwestern Greece.

Dry matter digestibility of species-rich rangelands provides a synthetic measure of the amount of energy in plant constituents available for ruminants and it is a crucial factor for the estimation of nutritive value of rangelands (Bruinenberg et al., 2002). In vitro dry matter digestibility showed a trend to decrease progressively from spring to autumn in all three grasslands. As referred by Van Soest (1994), lignin is a principal factor limiting digestibility. In addition, the low protein and the high fiber contents have negative effects on digestibility (Minson, 1982). In all cases, the highest IVDMD value was reached when the fiber contents were the lowest (at the beginning of the growing season). These results are consistent with those of Mountousis et al. (2008), who indicated that monthly variations in dry matter digestibility are mainly related to those of crude protein and fiber contents. Additionally, according to Moreira et al. (2004), an increased leaf ratio in forage results in an increase in CP content and a reduction in cell wall content, which results in an increase in the digestibility of the forage.

### 4.3 Mineral concentrations

Forage is the main source of minerals for grazing animals. Mineral concentration of forage may vary among different grassland and perhaps within each of them (Márquez-Madrid et al., 2017). Phosphorous and calcium are the most important nutrients (McDonald et al., 2010) due to their role in the metabolic functions of livestock (Underwood and Suttle, 1999). Calcium requirements for growing cattle of 200–250 kg and small ruminants of 50 kg are in the range of 3.0 and 2.0 g kg${}^{-1}$ DM, respectively (NRC, 1985, 1996). In our study, herbage Ca concentration was found to be adequate and sufficiently higher than those normally required by the ruminants in all three grasslands. Additionally, increased phosphorus availability in soil leads to higher grassland dry matter productivity (Melo et al., 2007). In accordance with Ndebele et al. (2005), the results of the present study revealed that the phosphorus concentration of herbaceous plants declines markedly with advancing maturity. Phosphorus is the most deficient mineral for grazing livestock throughout the world according to Greene (2000). The estimated maintenance requirements for beef cattle and small ruminants are 1.7 and 1.6 g kg${}^{-1}$ DM, respectively (NRC, 1985, 1996). In our study, phosphorus content was enough to cover the maintenance requirements of small ruminants and beef cattle for the greater period only in the mountainous grasslands. However, phosphorus supplements are necessary, especially after June.

### 4.4 Animals' welfare

The welfare of animals is a quite new scientific multidimensional field which combines the practicing of animal husbandry with natural, social, ethical, cultural, economic and political issues (Mikuš et al., 2018). Even though there are common guidelines and directives for the animal welfare policy, little attention has been given to extensive production systems (Turner and Dwyer, 2007). An important aspect in the assessment of grazing ruminants' welfare is the free selection of an adequate and nutritious amount of feed from pastures. In our study, the higher stocking density (0.92 LU ha${}^{-1}$) of the semi-mountainous grassland compared to the other two grasslands is not a restrictive parameter in animals' welfare as it is beyond the appropriate limit (National Statistical Service of Greece, 2003). Generally, stocking density in Greece is not uniformly distributed over the country (Yiakoulaki and Papanastasis, 2014), but it is higher in the lower elevation zone, near the villages and in animal concentration points, such as watering troughs and sheds. Given the detected nutrient deficiencies, protein and phosphorus supplementation should be provided to animals in order to cover their maintenance requirements during the whole period and reach high levels of welfare. Nutrient deficiencies in animals should be timely identified and prevented before the clinical signs appeared.

Good grazing animals' welfare also requires free access to water, shelter for protection against the extreme climatic conditions, appropriate veterinary care, more human–animal contact and low-stress handling (Goddard et al., 2006; Turner and Dwyer, 2007). The studied grasslands as heterogeneous landscapes with rough topography and steep slopes are usually characterized by a lack of infrastructure, such as road networking, watering places, shelters, salt placement, strategic fencing, etc. Water, salt, mineral and protein supplements can be used by farmers as attractants to improve the livestock distribution and grazing uniformity on grasslands and to achieve higher levels of welfare. To improve awareness of farmers for the grazing animals' welfare problems, further studies are necessary to understand their knowledge and attitudes on this issue.

## 5 Conclusions

The determination of forage production and nutritive value in grasslands is important for achieving the optimum productivity of grazing livestock and their welfare. The studied grasslands provided to grazing animals high-quality feed during spring but their quality is declining steadily over time. The cell wall constituents generally increased, while the CP content and IVDMD decreased, leading to a decline in the overall nutritive value. From the mineral perspective, calcium was adequate to meet the grazing ruminants' requirements during the whole study period, while, on the contrary, a phosphorus supplementation is needed especially after June. Moreover, considerable monthly and interannual variations in the herbage production and nutrient content of the three grasslands were detected. The sub-alpine grassland was the most productive grassland with the highest mean CP content. Taking into account that in this grassland, high herbage production and quality are available during summer, the moving of animals from the semi-mountainous and mountainous regions to the sub-alpine zone seemed to be a proper practice for the better and long-term utilization of the studied grasslands. Monthly and interannual variations of forage availability and nutritive value in grasslands, as well as the lack of infrastructure, may have a negative effect on grazing ruminants' welfare.

## Data Availability

The original data are available upon request from the corresponding authors.
